# Direct-to-consumer strategies to promote deprescribing in primary care: a pilot study

**DOI:** 10.1186/s12875-022-01655-5

**Published:** 2022-03-22

**Authors:** Amy M. Linsky, Nancy R. Kressin, Kelly Stolzmann, Jacquelyn Pendergast, Amy K. Rosen, Barbara G. Bokhour, Steven R. Simon

**Affiliations:** 1 Section of General Internal Medicine, VA Boston Healthcare System, Boston, MA USA; 2 Center for Healthcare Organization and Implementation Research, VA Boston Healthcare System and VA Bedford Healthcare System, Boston, MA USA; 3 Section of General Internal Medicine, Boston University School of Medicine, Boston, MA USA; 4 Department of Surgery, Boston University School of Medicine, Boston, MA USA; 5 Department of Population and Quantitative Health Sciences, University of Massachusetts Chan Medical School, Worcester, MA USA; 6 Center for the Study of Healthcare Innovation, Implementation and Policy, VA Greater Los Angeles Healthcare System, Los Angeles, CA USA; 7 David Geffen School of Medicine, University of California Los Angeles, Los Angeles, CA USA

**Keywords:** Deprescriptions, Inappropriate Prescribing, Decision Making, Shared, Patient Participation

## Abstract

**Background:**

Deprescribing, or the intentional discontinuation or dose-reduction of medications, is an approach to reduce harms associated with inappropriate medication use. We sought to determine how direct-to-patient educational materials impacted patient-provider discussion about and deprescribing of potentially inappropriate medications.

**Methods:**

We conducted a pre-post pilot trial, using an historical control group, at an urban VA medical center. We included patients in one of two cohorts: 1) chronic proton pump inhibitor users (PPI), defined as use of any dose for 90 consecutive days, or 2) patients at hypoglycemia risk, defined by diabetes diagnosis; prescription for insulin or sulfonylurea; hemoglobin A1c < 7%; and age ≥ 65 years, renal insufficiency, or cognitive impairment. The intervention consisted of mailing medication-specific patient-centered EMPOWER (Eliminating Medications Through Patient Ownership of End Results) brochures, adapted to a Veteran patient population, two weeks prior to scheduled primary care appointments. Our primary outcome – deprescribing – was defined as clinical documentation of target medication discontinuation or dose-reduction. Our secondary outcome was documentation of a discussion about the target medication (yes/possible vs. no/absent). Covariates included age, sex, race, specified comorbidities, medications, and utilization. We used chi-square tests to examine the association of receiving brochures with each outcome.

**Results:**

The 348 subjects (253 intervention, 95 historical control) were primarily age ≥ 65 years, white, and male. Compared to control subjects, intervention subjects were more likely to have deprescribing (36 [14.2%] vs. 4 [4.2%], *p* = 0.009) and discussions about the target medication (31 [12.3%] vs. 1 [1.1%], *p* = 0.001).

**Conclusions:**

Targeted mailings of EMPOWER brochures temporally linked to a scheduled visit in primary care clinics are a low-cost, low-technology method associated with increases in both deprescribing and documentation of patient-provider medication discussions in a Veteran population. Leveraging the potential for patients to initiate deprescribing discussions within clinical encounters is a promising strategy to reduce drug burden and decrease adverse drug effects and harms.

## Background

Inappropriate medication use, conceptualized as when a medication’s potential risk outweighs its potential benefit, can result from drug-drug interactions, inappropriate dosing, or duration of use longer than recommended [1]. Resulting in negative consequences, including patients’ experience of adverse drug events and associated sequelae, increased out-of-pocket expenses, and pill burden [2–4]. Harms from medications can lead to increased healthcare system utilization and overall costs [1, 5–7]. Efforts to reduce inappropriate medications traditionally have focused on reducing initial prescriptions (e.g., antibiotics for probable viral infections); [8] however, another effective approach is deprescribing [9, 10]. This strategy may be more widely applicable given the frequency with which inappropriate medications are currently used by patients [11, 12].

Deprescribing is the intentional discontinuation of medications, with the decision occurring within the context of a patient’s overall clinical status and integrating patients’ goals and values for healthcare treatment [10]. Potential benefits include improved medication adherence, enhanced patient satisfaction, and decreased costs to the patient and healthcare system [13, 14]. While there are few identified harms from discontinuing medications, and adverse drug- withdrawal events are rare, [15, 16] many patient and provider barriers to deprescribing preclude it from occurring as often as might be beneficial in clinical practice [17–21]. Patients may have concerns about symptom return or may be reluctant to change medications, especially in the context of multiple clinicians or having been instructed about the importance of medication adherence. Meanwhile, providers often have limited time or feel pressures to meet performance metrics that disincentivize deprescribing, such as achieving strict hemoglobin A1c (HbA1c) goals in patients with diabetes. As a result, efforts to facilitate deprescribing have included interventions targeting healthcare systems, providers, patients, and combinations thereof, (e.g., comprehensive medication review, provider education) with varying success [22, 23].

Including the patient in deprescribing interventions frequently yields increased action by providers to reduce medications. One mechanism to promote deprescribing is outreach targeted to patients, analogous to direct-to-consumer advertising for medication initiation, a strategy that increases appropriate and inappropriate prescribing [24]. The EMPOWER (Eliminating Medications Through Patient Ownership of End Results) brochures exemplify this patient-centered deprescribing approach, capitalizing on cognitive dissonance and adult learning theory to educate and activate patients to discontinue medications under the guidance of a healthcare provider [25, 26]. Direct-to-consumer mechanisms also reduce the onus on providers, who are frequently responsible for initiating clinical activities and are often the target of deprescribing interventions.

Two medication classes addressed by EMPOWER brochures are proton pump inhibitors (PPIs) and sulfonylureas. While PPIs are a potent and effective gastric acid reducing treatment for conditions such as Barrett’s esophagus, peptic ulcer disease, and gastroesophageal reflux disease (GERD), they are often used at higher doses or for longer duration than indicated [27, 28]. When used inappropriately, PPIs can needlessly increase drug burden and costs. Similarly, sulfonylureas successfully reduce hyperglycemia in patients with diabetes; however, relative to other treatment options, they also place patients at higher risk of hypoglycemia and its potential harms (e.g., falls, loss of consciousness, seizures), especially among older adults for whom guidelines recommend less aggressive treatment targets [29–33]. With the ultimate goal of preventing medication-associated harm, we sought to determine how medication-specific EMPOWER brochures, adapted to a Veteran patient population, impacted discussion about and deprescribing of potentially inappropriate medications (PIMs).

## Methods

### Intervention

We used EMPOWER brochures, designed by the Canadian Deprescribing Network [34]. These brochures provide detailed information about the medication, allow patients to test their knowledge of the medication and indications for use, reflect on their own experiences with potential side effects, discuss alternative therapies (medication and non-pharmacologic options), and include a vignette of a patient who successfully discontinued the medicine. These decision aids were written at a 6^th^ grade reading level and were based upon theories of patient activation (e.g., skills and confidence to enable patient engagement), adult learning (e.g., knowledge acquisition is immediately applicable), and cognitive dissonance (i.e., contradictory behaviors and/or beliefs) [25, 35–37]. The brochures repeatedly emphasize that patients should not make any medication changes without first consulting with their health care provider. We tailored both study brochures for the Veteran population; for example, we adapted images and only used generic medication names. We further expanded the sulfonylurea brochure to include insulin to align with the VA Hypoglycemia Safety Initiative efforts, an effort to reduce unnecessary medications, with a focus on insulins and sulfonylureas [38].

Subjects were identified weekly during the 3-month intervention window and mailed the applicable EMPOWER brochure. No additional contact was made with the subject prior to the index visit.

### Study setting, population, and design

**Fig. 1:**
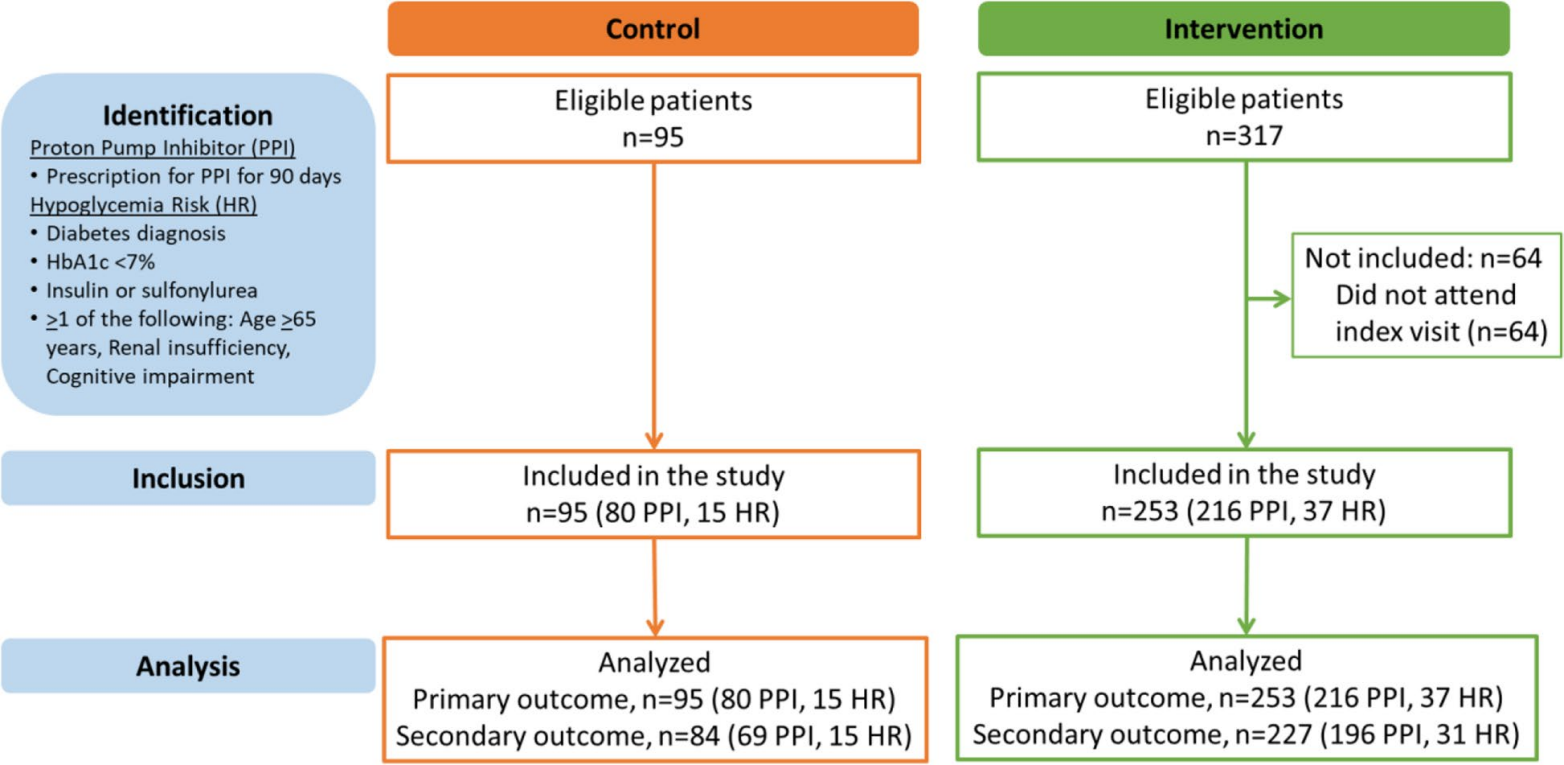
STROBE Flow Chart

We conducted a pre-post pilot study of a patient-centered intervention at one campus of an urban VA medical center (VAMC) (Fig. 1). For this trial, we identified eligible subjects who met inclusion criteria for one of two medication-based cohorts and were scheduled for appointments two weeks in the future (index visit from October 16, 2017 to January 14, 2018) in either Primary Care Clinics (*n* = 14 attending primary care providers [PCPs], 17 resident PCPs) or Women’s Health Clinics (*n* = 3 PCPs). We identified an historical control group of patients seen in primary care in the month prior to the intervention (September 18, 2017 to October 15, 2017) who met study eligibility criteria. These patients received usual care.

The first cohort comprised patients with hypoglycemia risk (HR) due to potential overtreatment of diabetes. Inclusion criteria were aligned with the VA Hypoglycemia Risk Initiative and required 1) a diagnosis of diabetes based on International Classification of Diseases, Tenth Revision, Clinical Modification (ICD-10-CM) codes, 2) most recent HbA1c < 7%, and 3) prescription for either insulin or sulfonylurea, as well as 4) one or more of the following factors associated with increased hypoglycemia risk: age 65 or older; renal insufficiency, defined as creatinine > 2 mg/dL; or cognitive impairment, defined by a diagnosis of cognitive impairment or dementia and/or treatment with an acetylcholinesterase inhibitor (e.g., donepezil).

The second cohort included patients of any age prescribed a proton pump inhibitor (PPI) at any dose for 90 consecutive days. We included patients who may have had an indication to remain on a PPI (e.g., diagnosis of Barrett’s esophagus, medications associated with peptic ulcer) because of 1) difficulty determining PPI appropriateness from administrative data (i.e., status and severity of conditions), and 2) our study procedures promoted discussion of deprescribing rather than directly discontinuing the medication.

Patients meeting inclusion criteria for both the hypoglycemia and PPI cohorts were placed in only the hypoglycemia risk cohort because the short-term risks from hypoglycemia are usually greater than those from chronic PPI use. Because we erred on the side of including patients who *might* be candidates for medication deprescribing, we acknowledge that the goal deprescribing rate would be less than 100%, since medication continuation would be appropriate for some study subjects. Subjects were eligible to participate in the study once, regardless of additional PCP visits during the study. Patients who did not attend the index visit were excluded from analyses.

Prior to the intervention window, PCPs received a one-time brief informational overview of the study, along with educational material (i.e., algorithm) about deprescribing the targeted medications.

### Study measures

We conducted a chart review of completed progress notes to identify our *primary outcome of deprescribing*, defined as the clinical documentation that the dose of the target medication was decreased or the medication was discontinued completely. In contrast, deprescribing was considered absent when the target medication dose was unchanged or increased. We also conducted chart reviews to assess our *secondary outcome,* namely the occurrence of patient-provider *discussion* about the target medication, operationally defined as documentation of discussion about the target medication (yes or possible vs. no or absent). While the discussion likely is proximal to the intervention, we selected deprescribing as our primary outcome given its reflection of clinical action.

Additional variables extracted from administrative data in the VA Corporate Data Warehouse included age; sex; race; and specified comorbidities, medications, and utilization in the year prior to or 20 days following the index visit that may have been an indication or contraindication for the target medication (e.g., use of antiplatelet agents may indicate a need for a PPI) [39].

### Analyses

To assess non-response bias (i.e., subject non-attendance at index PCP visit), we began by evaluating differences between the intervention subjects in our study population (i.e., received brochure and attended index visit) with those excluded from analyses (i.e., received brochure but did not attend their index PCP visit). We then examined patient characteristics and compared the intervention to control subjects, using descriptive statistics and chi-square tests.

We used chi-square tests to examine the association of receiving brochures with our primary and secondary outcomes of deprescribing and discussions, respectively. We assessed differences in deprescribing by PCP with one-way analysis of variance (ANOVA). We then conducted stratified analyses by medication cohort for the primary outcome, where we additionally examined patient characteristics associated with deprescribing.

All analyses were conducted with SAS statistical software, version 9.4.

## Results

### Population description

We mailed brochures to 317 potential intervention subjects; of these, 253 attended the index PCP visit and were included in the study population. There were no statistically significant differences between subjects who were included vs. excluded from analyses (data not shown), with the exception of age ≥ 65 years (80.5% of included subjects vs. 67.9% of excluded subjects; *p* = 0.037), receipt of gastroenterology procedure (7.3% of included subjects vs. 16.1% of excluded subjects; *p* = 0.03), and diagnosis of esophagitis (0.4% of included subjects vs. 3.6% of excluded subjects; *p* = 0.03).

The entire study population of 348 patients, which included the 253 intervention subjects and 95 control subjects, was predominantly 65 years of age or older, white, and male (Table 1). There were no statistically significant differences between the intervention and control subjects with respect to clinical providers (e.g., trainee or women’s health clinician) or select diagnoses and medication use related to the target medication. There were 296 subjects in the PPI cohort (80 control, 216 intervention), with a greater proportion of intervention patients having any gastrointestinal diagnosis (49.1% vs. 36.3%, *p* = 0.049). There were 52 subjects in the hypoglycemia cohort (15 control, 37 intervention), with intervention subjects having lower prevalence of renal impairment (13.5% vs 40.0%, *p* = 0.034) and lower frequency of visits to an endocrine clinic (18.9% vs. 46.7%, *p* = 0.041). Complete data were available for deprescribing outcomes; however, because not all progress notes were available for review, we could not determine for all subjects the occurrence of a patient-provider discussion about deprescribing the target medication (n missing = 30; 26 intervention [20 PPI, 6 HR], 4 control [4 PPI]).

**Table 1:** Characteristics of study population

	**Total Population (*n* = 348)**			**PPI Cohort only (*n* = 296)**			**Hypoglycemia Risk Cohort only (*n* = 52)**		
**Variable**^a^	**Control (*n* = 95)n (%)**	**Intervention (*n* = 253) n (%)**	***P*** **Value**	**Control (*n* = 80)n (%)**	**Intervention (*n* = 216) n (%)**	***P*** **Value**	**Control (*n* = 15)n (%)**	**Intervention (*n* = 37) n (%)**	***P*** **Value**
Age ≥ 65	73 (76.8)	204 (80.6)	0.43	59 (73.8)	167 (77.3)	0.52	14 (93.3)	37 (100)	0.29
White	75 (79.0)	205 (83.3)	0.34	63 (78.8)	177 (84.7)	0.23	12 (80)	28 (75.7)	1.0
Male	89 (93.7)	238 (94.1)	0.89	76 (95.0)	201 (93.1)	0.79	13 (86.7)	37 (100)	0.08
PCP is a resident	10 (10.5)	30 (11.9)	0.73	8 (10.0)	26 (12.0)	0.63	2 (13.3)	4 (10.8)	1.0
PCP is in Women’s Health Clinic	5 (5.3)	8 (3.2)	0.36	4 (5.0)	8 (3.7)	0.74	1 (6.7)	0 (0	0.29
Had a GI procedure	n/a	n/a	n/a	5 (6.3)	17 (7.9)	0.64	n/a	n/a	n/a
Was seen in a GI clinic	n/a	n/a	n/a	19 (23.8)	76 (35.2)	0.061	n/a	n/a	n/a
Diagnosis of GI condition	n/a	n/a	n/a	**29 (36.3)**	**106 (49.1)**	**0.049**	n/a	n/a	n/a
Glucocorticoid use^b^	n/a	n/a	n/a	3 (3.8)	19 (8.8)	0.21	n/a	n/a	n/a
NSAID use^b^	n/a	n/a	n/a	45 (56.3)	111 (51.4)	0.46	n/a	n/a	n/a
Clopidogrel use^b^	n/a	n/a	n/a	3 (3.8)	11 (5.1)	0.77	n/a	n/a	n/a
Insulin (rather than sulfonylurea) use	n/a	n/a	n/a	n/a	n/a	n/a	10 (66.7)	15 (40.5)	0.088
Diagnosis of renal Impairment (Creatinine > 2 mg/dL)	n/a	n/a	n/a	n/a	n/a	n/a	**6 (40)**	**5 (13.5)**	**0.034**
Diagnosis of cognitive impairment or dementia	n/a	n/a	n/a	n/a	n/a	n/a	1 (6.7)	5 (13.5)	0.66
Visit to an endocrine clinic	n/a	n/a	n/a	n/a	n/a	n/a	**7 (46.7)**	**7 (18.9)**	**0.041**
History of hypoglycemia	n/a	n/a	n/a	n/a	n/a	n/a	0	0	–

*PCP* Primary Care Provider, *PPI* Proton Pump Inhibitor, *GI* gastroenterology, *NSAID* non-steroidal anti-inflammatory drug

Bold indicates statistically significant results

^a^All diagnoses, clinic visits, medications, and procedures identified in the year prior to the index visit

^b^Indicates medications that may justify PPI prescriptions

### Deprescribing and discussion outcomes

**Fig. 2:**
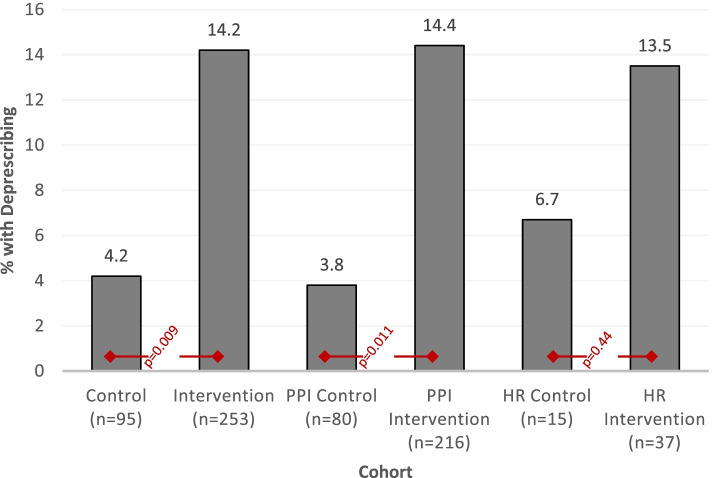
Deprescribing outcomes. *PPI* Proton Pump Inhibitor, *HR* Hypoglycemia Risk

Among the entire population of 348 subjects, *deprescribing* was more common among intervention subjects compared to controls, with an absolute magnitude difference of 10% (36 [14.2%] vs. 4 [4.2%], *p* = 0.009, Fig. 2). Deprescribing rates differed between PCPs (*p* = 0.03); however, there were no differences between resident clinicians compared to attending physicians (*p* = 0.83) nor between Women’s Health PCPs and general PCPs (*p* = 0.19) (data not shown). Deprescribing was more common among subjects who had a documented discussion about their medications compared to those who did not (17/32 [53.1%] vs. 20/280 [7.1%], *p* < 0.0001).

In stratified analyses (Fig. 2), the PPI cohort had similar findings, with more intervention than control subjects having their target medication deprescribed (31/216 [14.4%] vs. 3/80 [3.8%], *p* = 0.01). While there was a similar pattern of findings in the hypoglycemia cohort, differences were not statistically significant (5/37 [13.5%] intervention vs. 1/15 [6.7%] control, *p* = 0.44).

**Fig. 3:**
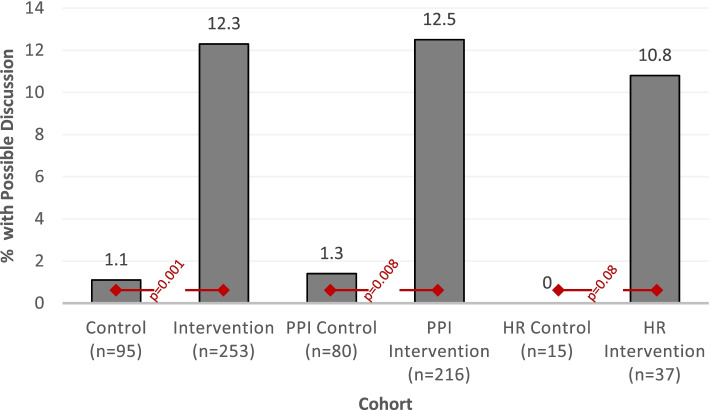
Discussion outcomes. *PPI* Proton Pump Inhibitor, *HR* Hypoglycemia Risk

Among the entire population, patient-provider *discussions* about the target medication were more common among intervention subjects compared to controls (31 [12.3%] vs. 1 [1.1%], *p* = 0.001, Fig. 3). In stratified analyses, the PPI cohort had similar findings, with more intervention than control subjects having discussions about their target medication (27/216 [12.5%] vs. 1/80 [1.3%], *p* = 0.008). While there was a similar pattern in the hypoglycemia risk cohort, differences were not statistically significant (4/37 [10.8%] intervention vs. 0/15 [0%] control, *p* = 0.08), likely due to the smaller sample size. Discussions were more often documented among subjects who had deprescribing (44% vs. 4.9%, *p* < 0.0001).

In stratified cohort analyses, deprescribing was more common in PPI subjects with a diagnosis of GERD compared to those without documented GERD (19.2% vs. 6.3%, *p* = 0.0006). No other patient characteristic in the PPI cohort, nor any patient characteristic in the hypoglycemia risk cohort, was associated with deprescribing.

## Discussion

Distributing EMPOWER brochures directly to patients in advance of an already-scheduled primary care visit is associated with increases in both deprescribing and documentation of patient-provider discussions of the target medication in a Veteran population. Given the common use of proton pump inhibitors and the frequency with which hypoglycemia-risk medications result in emergency department visits, a 10% absolute magnitude reduction in prescriptions at the population-level could potentially mitigate large numbers of adverse drug events and associated healthcare costs. In the case of PPIs, there is often a failure to ensure continued need and appropriateness; [40] patients in the PPI cohort with a diagnosis of GERD were more likely to have deprescribing, which may reflect inappropriate duration of use.

EMPOWER brochures have demonstrated success to reduce potentially inappropriate use of multiple medication classes. The EMPOWER trial, which addressed benzodiazepines, and the D-PRESCRIBE trial, which targeted sedative-hypnotics, first-generation antihistamines, glyburide, or nonsteroidal anti-inflammatory drugs, found community pharmacy-based distribution of these educational materials to older adults led to significant reduction in prescriptions for a potentially harmful medication in the subsequent six months [25, 26]. While we did not achieve the same absolute magnitude of deprescribing as these landmark trials, we used a different mechanism of information distribution, shorter follow-up time, and medications that may be viewed as having lower risk.

Our choice to send the material directly to patients highlights the value of providing patients with the knowledge and encouragement to be active participants in their health care decisions [41]. With increasing recognition of the importance of shared decision-making, [42] equipping patients to have thoughtful dialogue with their clinical providers is essential to providing patient-centered care [43]. The history of educational materials and decision aids to support thoughtful selection among equal options is well established for decisions across the healthcare continuum [44]. Acquisition of confidence and communication skills from medication-specific EMPOWER brochures may foster patients to remain active agents in other healthcare decisions, consistent with the theories upon which their development was based. However, clinicians must also possess the knowledge, skills, and attitudes to respond to patient-initiated discussions of clinical decisions. In the context of safe medication use, this competency includes knowledge of safe deprescribing. Multi-faceted efforts that include components to overcome provider-level barriers would likely have even greater success. For example, embedding pharmacists in a primary care setting to provide face-to-face medication reviews resulted in a 20% acceptance rate of pharmacist recommendations for deprescribing [45].

Shared decision-making regarding medications, diagnostic testing, and treatments for chronic or non-urgent conditions does not necessarily require an immediate choice. Decisions in these circumstances could – and perhaps should – occur over multiple visits and time to allow for patients to process the information and consult with family and friends. To that end, receipt of EMPOWER brochures may have initiated a chain of events that would subsequently lead to deprescribing via provision of knowledge. Longitudinal interactions with primary care and other clinicians provide multiple opportunities for discussion and decision-making [46]. While brochures in our study were temporally linked to a scheduled visit in Primary Care clinics, there may have been more urgent or higher priority needs at that visit, deferring deprescribing of the target medication to a subsequent encounter. Related, we do not know the quality or duration of the patient-provider relationship, nor prior attempts to deprescribe either the targeted or other medications. Understanding the effect of multiple opportunities to discuss and deprescribe on reduction of potential inappropriate medications would allow for identification of ideal timing of content delivery.

As evidence accumulates in support of EMPOWER brochures, the collection has expanded to include multiple different classes of medications [34]. The perception of danger or concern imparted with the brochure likely differs by medication class. Our choice of PPIs and medications with high risk of causing hypoglycemia may not be viewed by either patients or providers in the same manner they view benzodiazepines or other high-risk medications. While Veterans often obtain PPIs from the VA for cost reasons, their availability without a prescription potentially diminishes perceptions of risk [47]. On the other hand, the presence of performance metrics that evaluate providers on management of their patients with diabetes (e.g., glycemic control as measured by HbA1c) could disincentivize providers from reducing or discontinuing insulin or sulfonylureas, despite guidance for relaxed treatment targets [48–50]. Understanding the interaction of medication class with these complex decisions can yield more precise targeting of medications for which EMPOWER brochures foster deprescribing.

Several limitations should be noted. Because we mailed EMPOWER brochures to patients, we were unable to determine whether patients reviewed the information; however, this delivery mode was intentionally selected to minimize any burden on clinic staff or providers. Our outcome assessment was conducted by chart review, which was hindered by incomplete notes and poor documentation, as would be expected in any healthcare system. It is possible that patients were verbally instructed to reduce or stop medications without the change reflected in the medication ordering section of the electronic health record. Similarly, written progress notes may have omitted documentation of discussions. Conversely, if the provider took the time and effort to document a clinic-based conversation (without the use of templated text or clinical reminders), it seems reasonable that this documentation accurately reflects provider perceptions of the activities of the primary care visit. Taken together, we likely had under-ascertainment of our outcomes, biasing our findings toward the null. We only assessed outcomes up to 4 weeks after the visit; a longer follow-up period may have identified additional deprescribing events for both the target and non-target medications, or, for those who had deprescribing, resumption of the target medication or initiation of other treatments for the condition. Future studies should evaluate outcomes after a longer time interval to better understand the stability of deprescribing actions. We were unable to determine whether and to what degree the brief provider educational overview was associated with deprescribing, nor could we assess whether the patient or the provider initiated deprescribing discussions. Additional research is needed to better understand these aspects of the patient-targeted intervention, as well as issues related to implementation (e.g., feasibility). Our findings may not be generalizable to non-VA settings, as PPIs are lower cost in VA than when purchased over the counter (OTC), and guidelines from VA and the Department of Defense recommend relaxed glycemic targets compared to some professional societies [50]. However, the financial consequences for patients paying out-of-pocket for OTC PPIs may increase the effect of the EMPOWER brochures in non-VA settings.

## Conclusions

This single-site, time-limited pilot study illustrates the preliminary effectiveness of using direct-to-patient methods to promote deprescribing in VA settings. These promising associations can inform larger trials to further explicate the effectiveness and feasibility of targeted mailings of EMPOWER brochures temporally linked to a scheduled visit in primary care clinics. This low-cost, low-technology intervention can impart knowledge and leverage the ability of patients to drive change within clinical encounters and reduce prescribing of medications that may be unnecessary or place patients at risk. Discontinuing or reducing the dosage of risky medications has potential to reduce drug burden, decrease adverse drug effects and harms, and improve overall patient safety.

## Data Availability

The datasets collected and/or analyzed during the current study are available from the corresponding author on reasonable request.

## Abbreviations

DM: Diabetes Mellitus
EMPOWER: Eliminating Medications Through Patient Ownership of End Results
GERD: Gastroesophageal reflux disease
GI: Gastroenterology
HbA1c: Glycated hemoglobin
HR: Hypoglycemia Risk
HSR&D: Health Services Research and Development
ICD-10-CM: International Classification of Diseases Tenth Revision, Clinical Modification
OTC: Over-the-counter
PIM: Potentially inappropriate medication
PCP: Primary Care Provider
PPI: Proton pump inhibitor
VA: Veterans Affairs
VAMC: Veterans Affairs Medical Center
